# Comparative Embryology of Eleven Species of Stony Corals (Scleractinia)

**DOI:** 10.1371/journal.pone.0084115

**Published:** 2013-12-18

**Authors:** Nami Okubo, Takuma Mezaki, Yoko Nozawa, Yoshikatsu Nakano, Yi-Ting Lien, Hironobu Fukami, David C. Hayward, Eldon E. Ball

**Affiliations:** 1 Research and Education Center for Natural Sciences, Keio University, Yokohama, Kanagawa, Japan; 2 Kuroshio Biological Research Foundation, Hata, Kochi, Japan; 3 Biodiversity Research Center, Academia Sinica, Taipei, Taiwan, Republic of China; 4 Tropical Biosphere Research Center, Sesoko Station, Ryukyu University, Motobu, Okinawa, Japan; 5 Seto Marine Biological Laboratory, Field Science Education and Research Center, Kyoto University, Nishimuro, Wakayama, Japan; 6 Faculty of Agriculture, Miyazaki University, Gakuen-Kibanadai-Nishi, Miyazaki, Japan; 7 Evolution, Ecology and Genetics Group, Research School of Biology, Australian National University, Canberra, Australian Capital Territory, Australia; Pennsylvania State University, United States of America

## Abstract

A comprehensive understanding of coral reproduction and development is needed because corals are threatened in many ways by human activity. Major threats include the loss of their photosynthetic symbionts (*Symbiodinium*) caused by rising temperatures (bleaching), reduced ability to calcify caused by ocean acidification, increased storm severity associated with global climate change and an increase in predators caused by runoff from human agricultural activity. In spite of these threats, detailed descriptions of embryonic development are not available for many coral species. The current consensus is that there are two major groups of stony corals, the "complex" and the "robust". In this paper we describe the embryonic development of four "complex" species, *Pseudosiderastrea tayamai*, *Galaxea fascicularis*, *Montipora hispida*, and *Pavona Decussata*, and seven "robust" species, *Oulastrea crispata, Platygyra contorta, Favites abdita, Echinophyllia aspera, Goniastrea favulus, Dipsastraea speciosa* (previously *Favia speciosa*)*, and Phymastrea valenciennesi* (previously *Montastrea valenciennesi*). Data from both histologically sectioned embryos and whole mounts are presented. One apparent difference between these two major groups is that before gastrulation the cells of the complex corals thus far described (mainly *Acropora* species) spread and flatten to produce the so-called prawn chip, which lacks a blastocoel. Our present broad survey of robust and complex corals reveals that prawn chip formation is not a synapomorphy of complex corals, as *Pavona Decussata* does not form a prawn chip and has a well-developed blastocoel. Although prawn chip formation cannot be used to separate the two clades, none of the robust corals which we surveyed has such a stage. Many robust coral embryos pass through two periods of invagination, separated by a return to a spherical shape. However, only the second of these periods is associated with endoderm formation. We have therefore termed the first invagination a pseudo-blastopore.

## Introduction

Corals are highly variable in their reproductive patterns. There are species in which colonies may have a single sex throughout life, while other species may be sequential hermaphrodites, with sex based on size, or simultaneous hermaphrodites. They may also be synchronous spawners, releasing eggs and sperm or egg-sperm bundles, or brooders in which early development takes place within the colony and planulae are released. Considerably less is known about their patterns of early development, the topic of this paper.

Based on molecular sequence data, scleractinian corals can be divided into two large clades, the robust and the complex, that appear to have diverged more than 200MYA [1-5]. For many years, before the widespread availability of sequence data, coral taxonomy was based on morphological characters (e.g. 6,7) but few of the families created on this basis are presently regarded as monophyletic based on recent molecular studies [8]. Indeed, 8 of the 24 extant families recognized by Fukami et al. [2] have representatives in both the robust and complex clades [4]. Thus, the details of coral phylogeny are presently the subject of much debate, as is reflected in the title of a recent paper on the phylogeny of four coral families, which begins "Cleaning up the 'Bigmessidae' " [9]. Nevertheless, there appears to be general agreement on the existence of the two large clades mentioned above; the robust (so called because they are plate-like or massive) and complex (so called because they have diverse growth forms and are less heavily calcified), which were first proposed by Romano and Palumbi [1] on the basis of mitochondrial 16S ribosomal gene sequences. Figure S1 shows the species studied here mapped onto the phylogeny of Kitahara et al [3] and demonstrates that they are well distributed across the two major groups.

Because most corals spawn at night, and in some cases only once per year, descriptions of the development of many species are still fragmentary. This is in spite of the increasingly widespread interest in the group, which has developed as the threats posed by human activity have become recognized. This lack of knowledge is particularly acute for the earliest stages of development up to and including gastrulation, which are passed through quite rapidly.

Although there have been several reviews of coral reproduction and development in recent decades (e.g. 8,10-12) these have not dealt with the details of early development, which are found only in the primary literature, where the coverage is extremely patchy, with considerable detail available for some species and none for others. We here describe the early development of four complex corals; *Pseudosiderastrea tayamai, Galaxea fascicularis, Montipora hispida* and *Pavona Decussata* and seven robust corals; *Oulastrea crispata, Platygyra contorta, Favites abdita, Echinophyllia aspera, Goniastrea favulus, Dipsastraea speciosa* (previously *Favia speciosa*), *Phymastrea valenciennesi* (previously *Montastrea valenciennesi*: the last two genera were renamed in a recent taxonomic revision by Budd et al. [13]). As far as we are aware, development of the five species *P. tayamai, P. decussata, O. crispata, P. contorta*, and *E. aspera* has not previously been described in detail. We were particularly interested in whether formation of a flattened prawn chip stage early in embryonic development could be used as a diagnostic characteristic of a complex coral. 

## Materials and Methods

### Ethics statement

Research in Okinawa was carried out with permission of the Agriculture, Forestry and Fisheries Department of the Okinawa Prefectural Government for collecting adult coral colonies. No permit is required for collecting coral embryos in Okinawa. Permission numbers for *Montipora hispida* and *M. digitata* are "16-70", and for *Pseudosiderastrea tayami* and *Galaxea fascicularis* are "21-22". Elsewhere in Japan there are no laws either allowing or forbidding collection of adult corals or embryos. Embryos of *Pseudosiderastrea tayamai*, *Galaxea fascicularis*, *Montipora hispida* and *M. digitata* were collected in Okinawa Prefecture; embryos of *Platygyra contorta*, *Favites abdita*, *Echinophyllia aspera*, *Goniastrea favulus*, *Pavona Decussata* and *Phymastrea valenciennesi* (previously *Montastrea valenciennesi*) in Kochi Prefecture; embryos of *Dipsastraea speciosa* (previously *Favia speciosa*) and *Oulastrea crispata* in Wakayama Prefecture. We hope that this paper will help to make people aware of the importance of coral embryos in coral conservation.

### Embryo collection

Gametes of *Galaxea fascicularis* were collected from Sesoko Marine Biological Laboratory in Okinawa (26°63′ N, 127°86′ E); *Pseudosiderastrea tayamai* from Ogimi-son, Okinawa, Japan (26°70′ N, 128°12′ E); *Montipora digitata and Montipora hispida* from the reefs around Akajima Island, Okinawa (26°27′ N, 127°28′ E); *Favites abdita, Favites pentagona, Goniastrea favulus*, *Phymastrea valenciennesi* and *Pavona Decussata* from near the Laboratory of Kuroshio Biosphere Foundation, Kochi (32°78′ N, 132°73′ E); and *Dipsastraea speciosa, Echinophyllia aspera, Oulastrea crispata* and *Platygyra contorta* from Tanabe Bay near Kyoto Field Science Center, Wakayama (33°69′ N, 135°33′ E). The dates of all of these collections are given in Table 1 along with additional locality, habitat and spawning data. In some species, colonies were brought to the laboratory where spawning occurred in tubs. Once spawning had occurred, gametes were gently stirred to mix the bundles and ensure insemination. For these species the number of colonies involved in the crosses is given in brackets. In other species gametes were collected by divers on SCUBA from the surface of colonies as they spawned. These species are marked with an asterisk and in all cases 3 or more colonies were sampled. For all species, fertilized eggs were placed into 2.5L containers in filtered sea water and development allowed to proceed. Gametes and the early stages of development were sampled and examined hourly for the first 24 h after spawning and every 4 or 6 h thereafter. Typically, each container held more than 1000 embryos and at least three containers were set up for each species. In the case of *Pseudosiderastrea*, fewer embryos were obtained; in this case each of three containers held approximately 500 embryos. For observation, approximately 50 eggs or embryos were placed in a 75-mm Petri dish under a light microscope after which they were fixed for histology. The water temperature was maintained at 26.0 to 26.5°C throughout the period of observation and culture. A few days before the predicted day of spawning a small fragment was taken from colonies of *Montipora hispida*, and the timing of *Symbiodinium* entry was established by dissection. 

**Table 1:** Locality, habitat and spawning data for the species studied.

Species (colonies sampled)	Site (Japan)	Habitat of the colony studied	Date D/M/Y	Time	Tidal range	Mode	Egg Diameter (um)
**Complex corals**							
*Pseudosiderastrea tayamai* (7)	Okinawa (Ogimi-son)	Intertidal muddy and, rocky shore, 0-5m	09/7/09-16/7/09	22:00	Middle tide	Hermaphrodite	500
*Galaxea fascicularis* (3)	Okinawa (Sesoko)	Lagoon, 3-5m	15/6/09	22:00	Neap tide	Gonochoric (egg bundle from female, pseudo-egg-sperm bundle from male)	450
*Montipora digitata*	Okinawa (Akajima)	Lagoon, 3-5m	25/5/05	20:45	Spring tide	Hermaphrodite (egg-sperm bundle)	350~400
*Montipora hispida* (3)	Okinawa (Akajima)	Lagoon, 3-5m	26/5/05	22:00	Middle tide	Hermaphrodite (egg-sperm bundle)	350~400
*Pavona Decussata* (≥3)*	Kochi	Rocky shore, 3-5m	01/8/10 20/8/12	04:30♂ 04:40♀	Neap tide	Gonochoric	150
**Robust corals**							
*Echinophyllia aspera* (≥3)*	Kochi	Rocky shore, 3-5m	09/7/07	20:30	Neap tide	Hermaphrodite (egg-sperm bundle)	400
*Dipsastraea* (*Favia*) *speciosa* (3)	Wakayama	Rocky shore, 3-5m	11/8/06	20:00	Spring tide	Hermaphrodite (egg-sperm bundle)	400
*Favites abdita* (≥3)*	Kochi	Rocky shore, 3-5m	09/7/07	20:30	Neap tide	Hermaphrodite (egg-sperm bundle)	400
*Goniastrea pectinata* (≥3)*	Kochi	Rocky shore, 3-5m	09/7/07	20:30	Neap tide	Hermaphrodite (egg-sperm bundle)	350
*Phymastrea* (*Montastrea*) *valenciennesi* (≥3)*	Kochi	Rocky shore, 3-5m	09/7/07	20:30	Neap tide	Hermaphrodite (egg-sperm bundle)	450
*Oulastrea crispata* (10)	Wakayama	Intertidal muddy and, rocky shore, 0-5m	13/8/09 09/7/13	23:00	Neap tide	Hermaphrodite (no bundle), Gonochoric	150
*Platygyra contorta* (≥3)*	Kochi	Rocky shore, 3-5m	09/7/07	20:30	Neap tide	Hermaphrodite (egg-sperm bundle)	375

Asterisks indicate that gametes were collected by SCUBA divers directly from corals *in situ*. Egg size data for *Pseudosiderastrea, Pavona, Dipsastrea* (*previous Favia*)*, Echinophyllia and Phymastrea* (*Montastrea*) appear not to have been reported previously, while those for the other species are within previously reported limits [22,25,27,29].

### Histology

In all species except *Oulastrea crispata* (0) and *Pseudosiderastrea tayamai* (<100) approximately 100 eggs or embryos were fixed at a time in 10% formalin/90% filtered seawater. Fixed samples were embedded in glycol methacrylate (Technovit 7100; Heraeus Kulzer GmbH, Germany) and sectioned at a thickness of 5-7 um using a microtome (Leica RM2125; Leica Microsystems). All sections were mounted on glass slides coated with gamma-aminopropyltriethoxysilane to increase adhesion of the sections, and were stained using methylene blue.

### Micrographs

Micrographs of both living and fixed embryos were adjusted with Adobe Photoshop to bring them to a relatively uniform appearance for presentation. However, all adjustments were global except in a few cases where parts of nearby embryos were erased in order to focus on the main topic of the figure. Also, in some cases embryos were placed on a more contrasting background using Photoshop to enable better visualization.

### Terminology

*Acropora* embryos form an extended, flattened cellular bilayer, the "prawn chip" which then transforms, by poorly understood mechanisms, into a sphere with a pore in the side. Cells, and perhaps formerly cellularized material such as lipid, become internalized during this process. The pore then fully closes, forming a smooth sphere with no external sign of a pore, before a second pore, which will ultimately become the mouth, opens. Closure of the first pore was referred to as gastrulation by Hayashibara et al. [14], Ball et al. [15], Hayward et al. [16] and Grasso et al. [17] and we will follow that usage here. If it is accepted that blastopore closure marks the end of gastrulation then any internal space appearing thereafter is the forerunner of the gastrovascular cavity. In *Acropora*, blastopore closure and the start of swimming coincide and mark a well-defined transition between the embryo and planula larva. In other genera, particularly those where the blastopore never closes, but merges imperceptibly into the oral pore, there is no such easy, universally agreed distinction.

## Results and Discussion (by Species)

Natural history and anatomical observations are here presented together for each species. Spawning data are summarized in Table 1. In the descriptions below times are given in hours after the first cleavage of a fertilized egg.

### Complex corals

#### *Pseudosiderastrea tayamai*

In spite of its unique mode of development, aside from the abstracts of talks, there is no information on the early development of *Pseudosiderastrea tayamai* available in English. Although *P. tayamai* is a simultaneous hermaphrodite, it is not known whether self- or cross-fertilization occurs in this species, nor whether egg/sperm bundles or separate eggs and sperm are released. Its reproduction resembles that of a brooder in that the fertilized eggs initially develop in association with their parent colony, rather than being immediately released into the plankton. Prior to spawning, the polyps release mucus which forms a net covering the whole colony, in which the released eggs are trapped and in which debris can lodge. Once they are released the eggs are fertilized within this mucus net and begin development, as described by Nakano [20,21].

**Figure 1:**
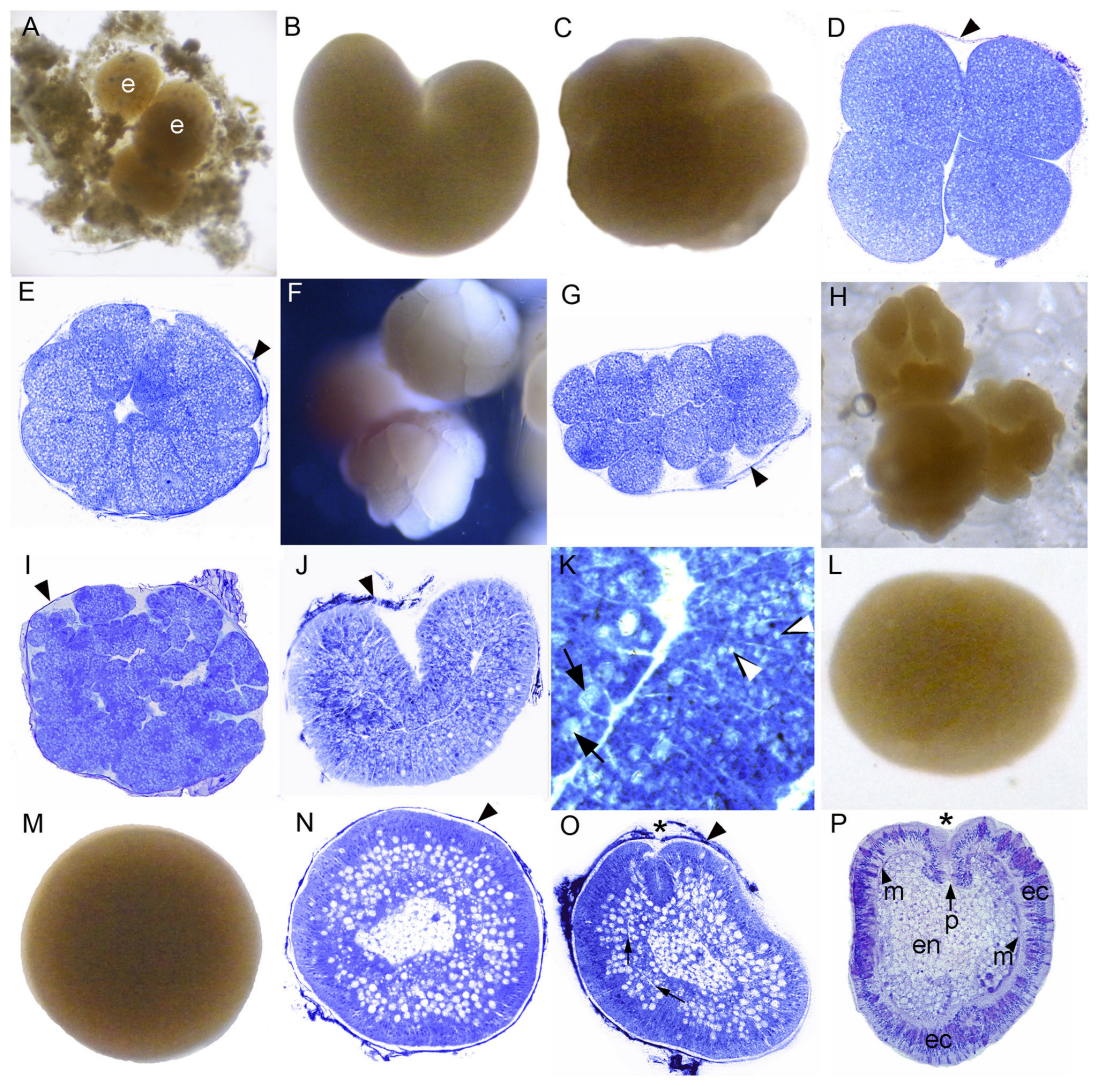
Development of *Pseudosiderastrea tayamai*. *Pseudosiderastrea tayamai* development is similar to that of *Acropora* *spp.* in that it passes through a stage consisting of a cellular bilayer lacking a central space. (A) Eggs (e) and debris enmeshed in the mucus net shortly after release. (B) 2-cell stage. (C) 4-cell stage. (D) A section of a 4-cell stage, with offset blastomeres. (E) Section of the 16 cell stage. (F) Compact spherical embryo. (G) Flattened embryo. (H) Intact embryos resembling a tightly cupped hand. (I) Section of an embryo similar to those in H, which if unwound would resemble a prawn chip. (J) A bowl-shaped embryo; (K) Enlargement of Figure 4J. Lipid bodies (white arrowheads) are gradually coalescing to form larger masses of lipid as they move centrally (black arrows). (L) The embryo forms a flattened sphere. (M) Later the embryo becomes more rounded. (N) Section of a spheroidal embryo. The lipids are moving centrally, out of the future ectoderm at the periphery and into the central future endoderm. (O) Section of a pear-shaped planula. Mesoglea formation is apparent between ectoderm and endoderm (arrows) and invagination has started (asterisk). Most of the lipids are in relatively large droplets but lipids are still present in the ectoderm. (P) In this elongate planula the pharynx (p) has formed, leading inward from the oral pore. Diverse cell types (e.g. nematocysts, granular cells) are now apparent in the ectoderm (ec), all lipids have moved into the endoderm (en), and the mesoglea (m) is clearly apparent separating endoderm from ectoderm. Traces of the mucus net are apparent surrounding many of the embryos (black arrowheads), even after histological processing.

This species has been reported to spawn every 2–3 weeks in May–August [20]. One hour after spawning, a portion of the mucus-debris net, with its entangled embryos, was placed in a plastic cup for culturing, where it sank to the bottom (Figure 1A). Early cleavage proceeded (Figure 1B-D) and each embryo formed a compact spherical mass (Figure 1E,F), within which the blastomeres remained firmly attached to each other, in contrast to comparable stages of the other coral species shown here. The next few stages were somewhat variable in shape, ranging from flattened (Figure 1G) to folded (Figure 1H,I). If an embryo such as that shown in Figure 1I could be unwound and flattened it would be seen to be organized into two layers, and to resemble an *Acropora* prawn chip. Next, the cells elongated, increasing the thickness of the walls of the bowl-shaped embryo (Figure 1J,K). This bowl-shaped structure then swelled to form a flattened sphere (Figure 1L), which rounded up with further development (Figure 1M,N). At this stage lipid bodies were still abundant (Figure 1N), but we are uncertain how to interpret their distribution. From Figure 1P it is clear that the center of the mature planula is filled with endoderm while the ectoderm is lipid free. So, the most parsimonious explanation of Figure 1N and 1O is that the central area constitutes endoderm that will grow in volume as lipid migrates from the future ectoderm. In planulae, such as that shown in Figure 1P, endoderm (en) and ectoderm (ec) are clearly demarcated by mesoglea (m) and a pharynx has formed, leading inward from the oral pore. Planulae such as that shown in Figure 1P remained motionless on the substratum, some forming a temporary mucus attachment to it. Then on the third day after spawning, they started swimming strongly, and left the mucus net.

#### *Galaxea fascicularis*

**Figure 2:**
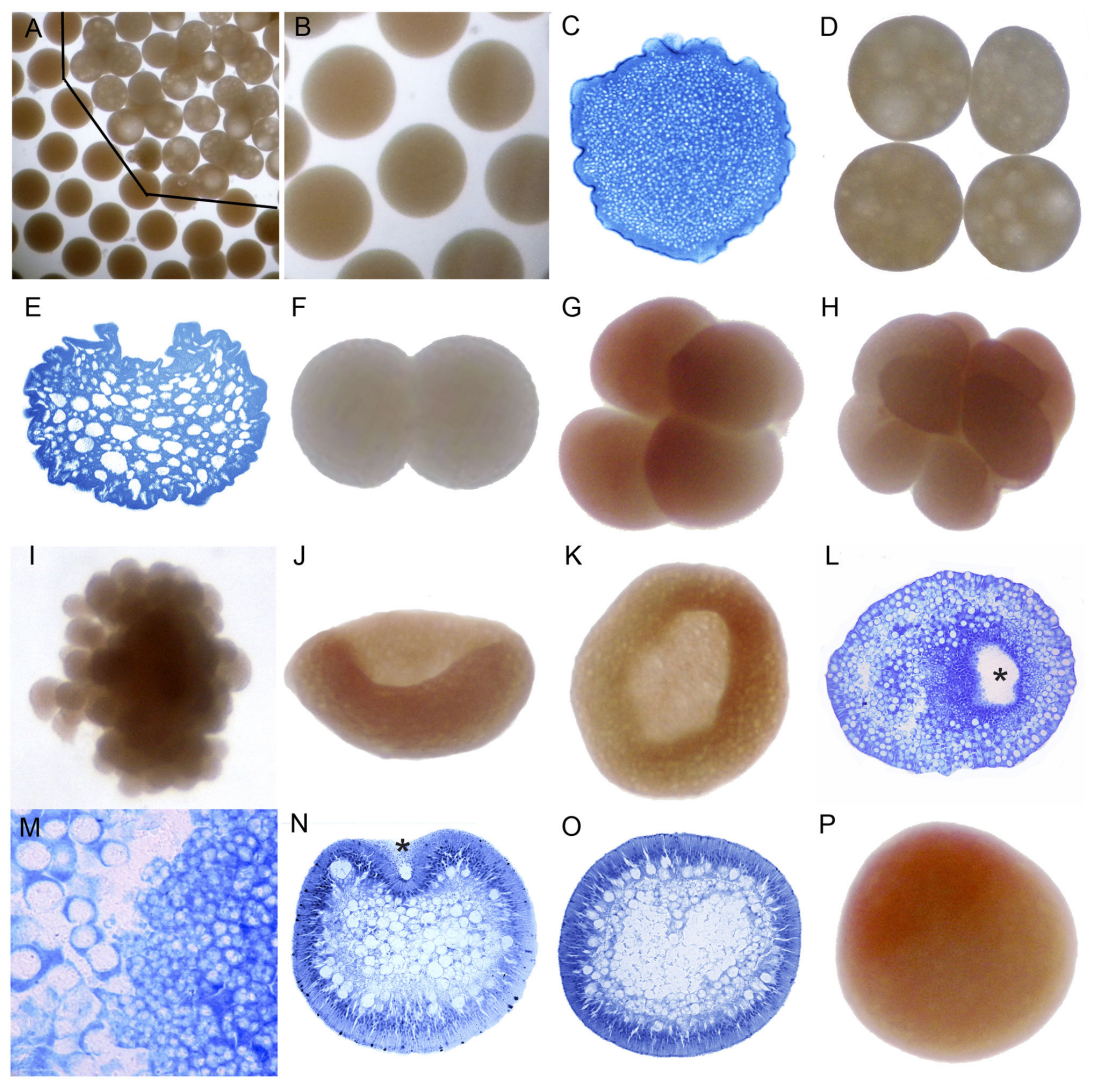
Development of *Galaxea fascicularis*. (A) Eggs (pinkish orange, below the line in the lower left corner) and pseudo-eggs (lighter in color, above the line, upper right corner). (B) True eggs are featureless and appear more dense and (C) a section reveals that they are filled with small lipid bodies. In contrast, pseudo-eggs appear vacuolated (D) and the lipid is localized to much larger bodies (E). (F) 2-cell stage. (G) 4-cell stage. (H) 8-16 cell stage. (I) Morula stage. (J) Bowl stage (concave side up). (K) Bowl stage (embryo shown in J-viewed from above). (L) Glancing section of an embryo comparable to J and K. (M) Enlargement of the embryo shown in L, with two sizes of lipid-containing bodies. It appears that the large arise by fusion of the small. (N) Spheroidal embryo with closing blastopore showing center filled with lipid. (O) Mesoglea is gradually forming at this stage. (P) Whole mount of stage similar to O. Red pigment localized at the animal pole as the result of a marking experiment persists in G, H, J, K and P. The asterisk in L and N marks the blastopore.

In Okinawa ([22], present study), the Australian Great Barrier Reef [23] and Taiwan [24] *Galaxea fascicularis* has a unique pattern of reproduction in which female colonies produce red eggs and hermaphroditic colonies produce sperm and white eggs (pseudo-eggs) which are spawned as egg-sperm bundles and are more buoyant than true eggs because of their greater content of wax-ester (Mita and Okubo, unpublished) (Figure 2A-E). The eggs have a natural reddish hue, but the more intense red apparent in Figure 2G, H, J, K and P is due to Neutral Red dye applied with a needle to cells at the animal pole earlier in development. There are conflicting reports on whether the white eggs are viable but we cannot specifically comment on this as we did not segregate the two sorts of eggs, having studied developing embryos, regardless of their source. 

Early cleavage is holoblastic (Figure 2F-H), despite the abundance of yolk granules. After approximately 10 hours, during which cleavage proceeded, the embryo assumed a concave-convex dish shape (Figure 2J,K; see also 24). The embryo then gradually thickened and became spherical, enclosing a core packed with yolk-containing cells (Figure 2N,O). The larvae started swimming after approximately 18 hours, while still spherical (Figure 2P).

#### *Montipora hispida* and *M. digitata*

Hirose and Hidaka [25] have previously provided a detailed, well-illustrated description of *M. digitata* embryonic development with a focus on the *Symbiodinium* and their transmission and location. While there is some variability in the distribution of *Symbiodinium* in individual embryos, we are in general agreement with their account. We here present data for *M. hispida*, which develops in a similar fashion.

**Figure 3:**
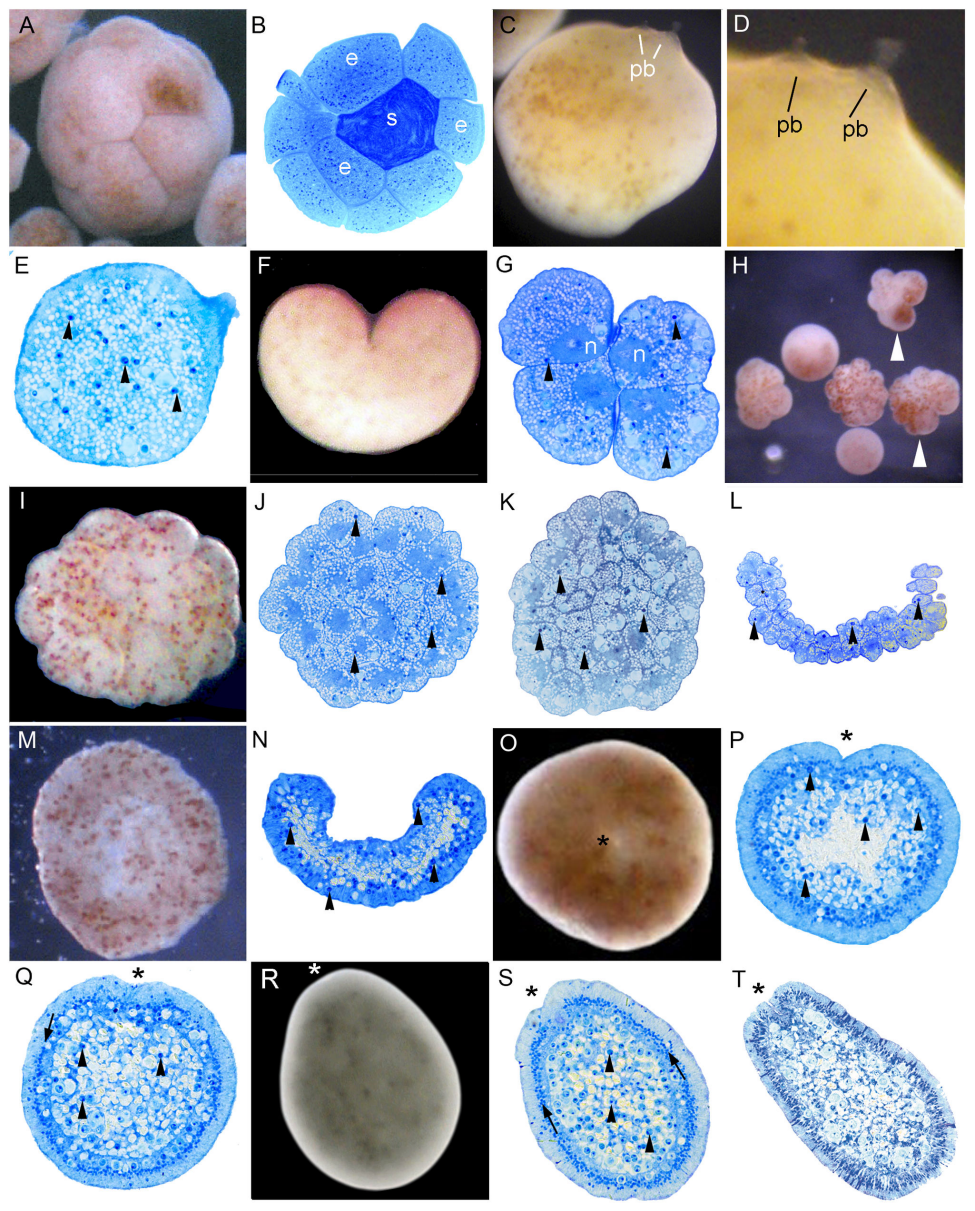
Development of *Montipora hispida*. (A) Egg-sperm bundle; *Symbiodinium* (darker spots within the eggs) are distributed throughout the eggs. (B) Section of egg-sperm bundle, showing sperm (s) tightly packed in the middle of the bundle, surrounded by eggs (e). (C) Polar bodies (pb) on the surface of the egg. (D) Enlargement of Figure 3C; (E) Section of egg. *Symbiodinium* are marked with arrowheads in this and succeeding panels. (F) The first cleavage results in a heart-shaped embryo. (G) Four-cell stage; the nucleus occupies a substantial part of each cell. (H) Embryos at the 4-16 cell stage, showing unevenly dispersed *Symbiodinium* in the arrowed embryos. (I) The 32-64 cell stage; again with *Symbiodinium* unevenly dispersed near the surface of the embryo. (J) Section of an embryo comparable to I. (K) Section of slightly older embryo. (L) Section of prawn chip stage. (M) Bowl-shaped stage, viewed from above. (N) Section of stage comparable to M, the embryo has become thicker. (O) Spherical stage, the asterisk in this and the following panels indicates the blastopore. (P) Section of an embryo similar to that shown in O. At this stage the mesoglea is gradually forming and *Symbiodinium* are moving into the endoderm. (Q) Later spherical stage; the mesoglea has clearly formed, with a row of ectodermal nuclei just above it (arrow). Few *Symbiodinium* are now seen in the ectoderm. (R) Pear-shaped planula stage. (S) Section of embryo comparable to that shown in R. There is a line of nuclei located in the columnar ectodermal cells, just above the mesoglea (arrows). (T) Section of elongated planula; the ectodermal cells are variously differentiated and the pharynx has grown inward.

**Figure 4:**
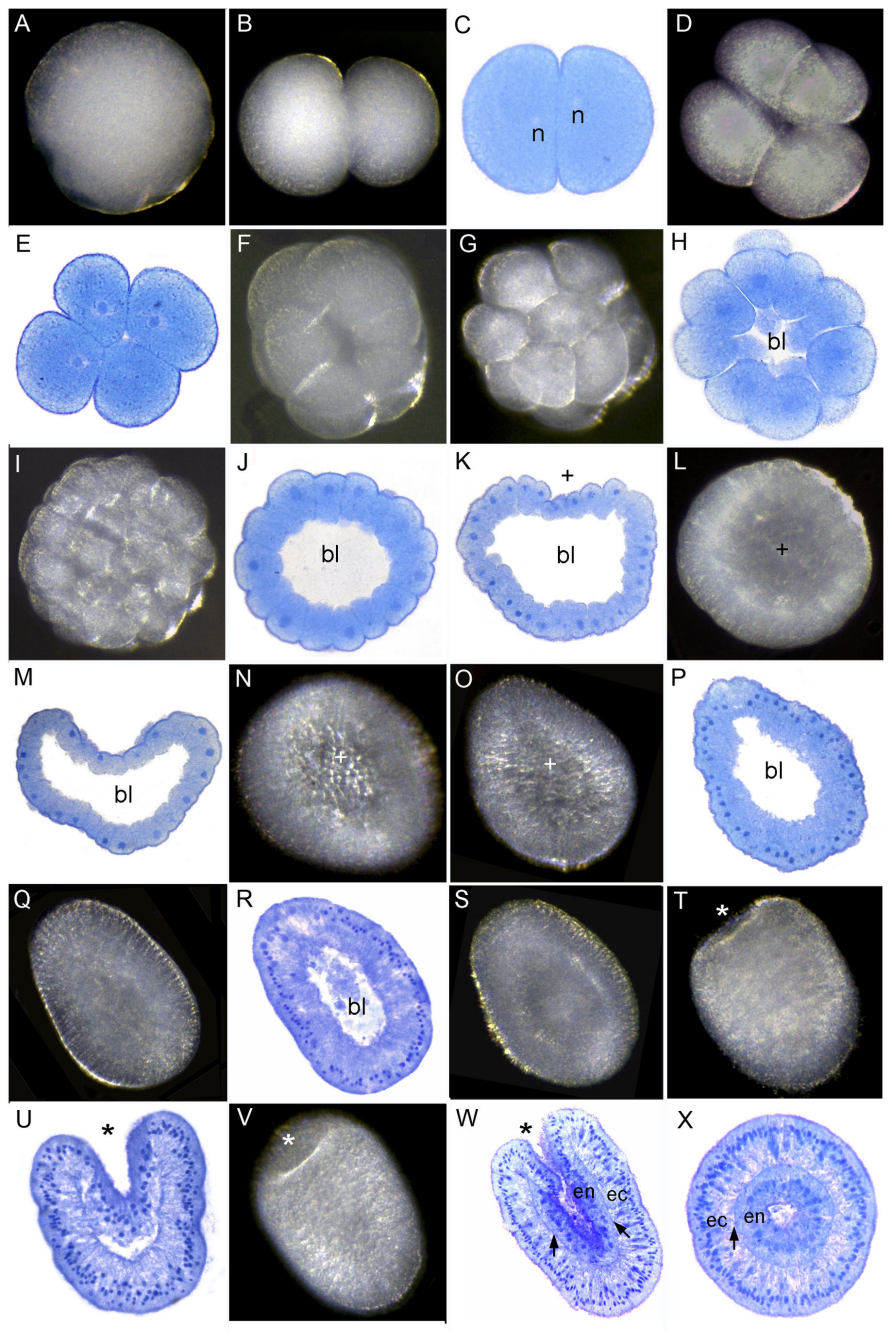
Development of *Pavona* *Decussata*. (A) Spawned egg. (B) 2-cell stage. (C) Section of 2-cell stage; the two nuclei are offset. (D) 4-cell stage. (E) Section of 4-cell stage. Successive divisions create additional blastomeres (F-G) 16-cell stage. (H) Section of 16-cell stage showing the start of the blastocoel (bl in this and subsequent figures). (I) 32-cell stage. (J) The cells surrounding the blastocoel are starting to become columnar. (K) The embryo starts to flatten and the first sign of the pseudo-blastopore appears (plus sign in this and subsequent panels). (L) The surface of the embryo becomes smoother due to continuing cell division. (M) Bowl-shaped embryo. (N-P) The embryo becomes pear-shaped, as the ectodermal cells become more elongate. (Q) Spheroidal blastula. (R) Section of the same, showing the blastocoel starting to fill as material moves inward. (S) Embryo just before appearance of the blastopore/oral pore. (T) Invagination of the blastopore/oral pore has begun (asterisk in this and subsequent panels). (U) Section of invaginating planula. (V) More elongate whole mount planula. (W) Invagination has proceeded, mesoglea has formed (arrows), and ectoderm (ec) and endoderm (en) are clearly apparent. (X) Transverse section of planula similar to W.

In *M. hispida*, starting from 5 days before the anticipated spawning, the gonads from 6 colonies were periodically dissected and observed until spawning. Three of these colonies spawned and in each of these *Symbiodinium* had appeared in the eggs between 8 and 32 hours before the evening spawning. The other three colonies, in which no *Symbiodinium* were observed within eggs, did not spawn until the following month. In both *M. digitata* and *M. hispida* egg-sperm bundles, in which the eggs surround a packet of sperm (Figure 3A,B), were released, as is also the case in *Montipora capitata* [26] and *Acropora* *spp.* [19]. This packaging results in sperm being carried to the surface by the buoyant eggs. Polar bodies were observed on the surfaces of eggs of *M. digitata* and *M. hispida* (Figure 3C,D). The area of the protrusion in which the polar body appears lacks lipid and *Symbiodinium* (Figure 3E). Two polar bodies were seen emerging near each other (Figure 3C,D) as previously observed by Babcock and Heyward [27] in *Goniastrea favulus*. The first cleavage resulted in two equal blastomeres (Figure 3F). The second cleavage occurred 1 hour after the first (Figure 3G), and the 16 cell stage another hour later. Distribution of *Symbiodinium* differed between individual blastomeres (Figure 4H, arrowheads). Cleavage proceeded (Figure 3I,J) and after approximately 7 hours the embryo reached the prawn chip stage (Figure 3K-M). The surface of the embryo gradually became smooth after about 11 hours due to continued cell division (Figure 3N), and the embryo gradually became spherical after 26 hours (Figure 3O). *Symbiodinium* began to concentrate in the endoderm (Figure 3P) after about this time. The blastopore, which had formed by 20 hours, remained open from this stage, eventually becoming the oral pore (asterisk, Figure 3O-T). This is in contrast to the situation in *Acropora*, where the blastopore closes. The embryos started swimming after about 33 hours. Mesoglea started to form (Figure 3Q,R) and the early larva became pear-shaped (Figure 3S). By this stage a majority of the *Symbiodinium* had moved to the endoderm, leaving only a few in the ectoderm. By the stage shown in Figure 3T the larva had elongated, the pharynx had developed, and strong swimming behavior was exhibited. 

#### *Pavona Decussata*

*Pavona Decussata* is gonochoric and releases sperm and sinking eggs, as is the case for *Pavona varians* outside of the Equatorial Eastern Pacific, where it is a reported to be a sequential hermaphrodite [12]. The first cleavage resulted in equal blastomeres (Figure 4A-C) with their nuclei offset. Cleavage continued (Figure 4D-G). By the stage shown in Figure 4H a blastocoel was apparent, which had expanded considerably after 3 hours (Figure 4H-J). Then, as cell division continued, a depression developed in one side after approximately 4 hours and became deeper for the next several hours (Figure 4K-N). The formation of an initial concavity unaccompanied by inward cell movement is a common feature of the robust corals and we refer to this concavity as a "pseudo-blastopore". The embryo then elongated to a pear-shape after 8-9 hours (Figure 4O-P), as the cells also elongated (Figure 4P). By 10 hours the embryo had become more spheroidal (Figure 4Q) and after approximately 12 hours swimming started, indicating that cilia had formed. At about this time some ectodermal nuclei had begun to migrate basally and material began to move into the blastocoel (Figure 4R). At approximately 20 hours, the aboral end became thicker (Figure 4S) and invagination started at the oral end (Figure 4T,U). Invagination proceeded (Figure 4V), and two germ layers, ectoderm (ec) and endoderm (en), were formed, separated by an obvious mesoglea (Figure 4W,X, arrows), which appears during the planula stage.

### Robust corals

#### *Oulastrea crispata*

**Figure 5:**
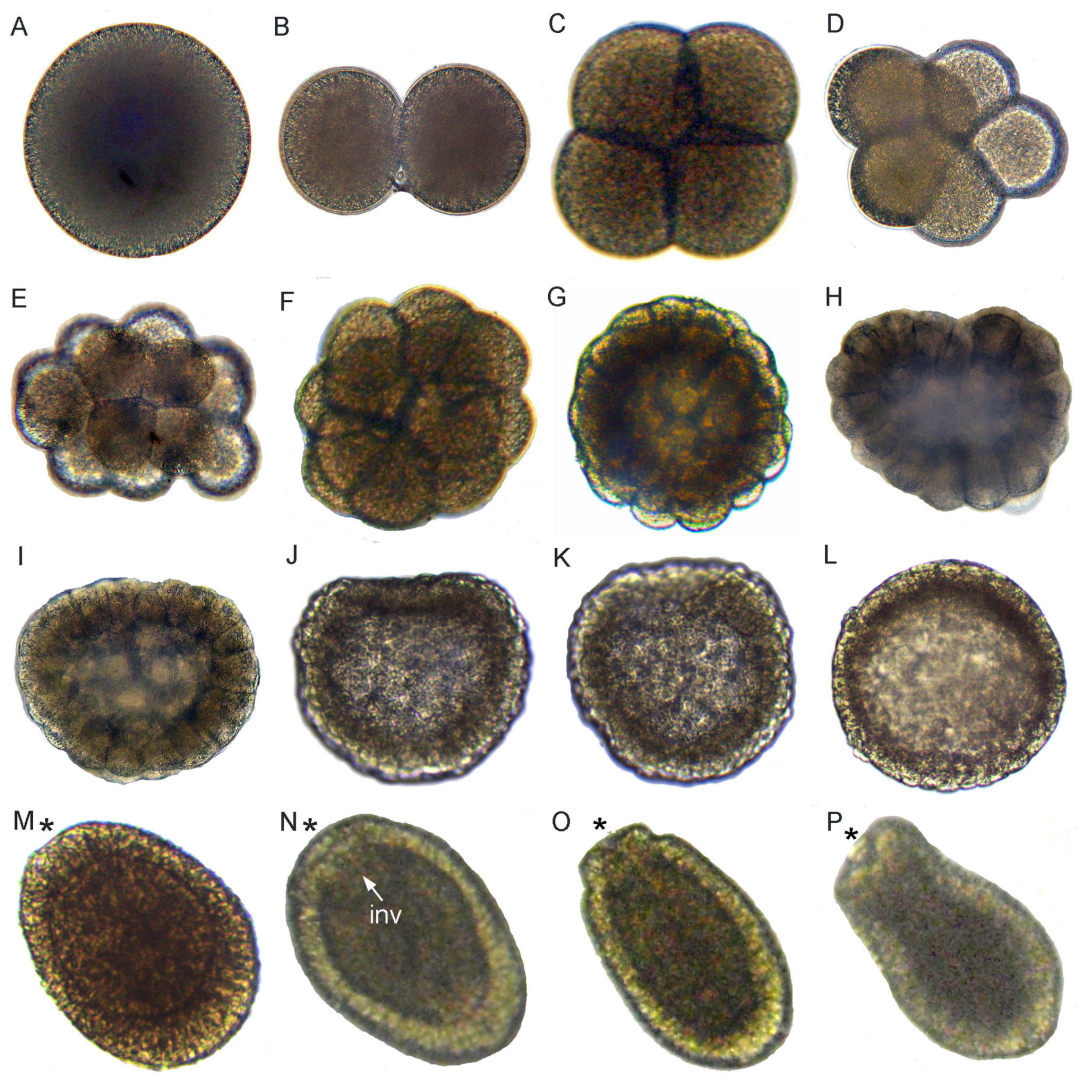
Development of *Oulastrea crispata*. (A) Spawned egg. (B) 2-cell stage. (C) At the 4-cell stage the blastomeres are offset. (D) 8-cell stage. (E) 16-cell stage. (F) Another embryo at the 16 cell stage. (G) This embryo has started to flatten. The blastocoel is apparent by the lightening at the center of the embryo. (H) By this stage the ectodermal cells are becoming columnar rather than circular in outline. The central blastocoel is apparent. (I-L) As cell division continues the flattened spheroid gradually resumes a spherical shape. (M) Pear-shaped planula. (N) Invagination (inv) has started at the oral end of the planula. (O) There is a ledge-like constriction at the oral end of the planula and the aboral end has thickened in preparation for settlement. (P) The constriction at the oral end of the planula is now less sharp and has moved aborally, the aboral end has become more rounded and the oral pore is now clearly apparent. In M-P the blastopore/oral pore is marked with an asterisk.

**Figure 6:**
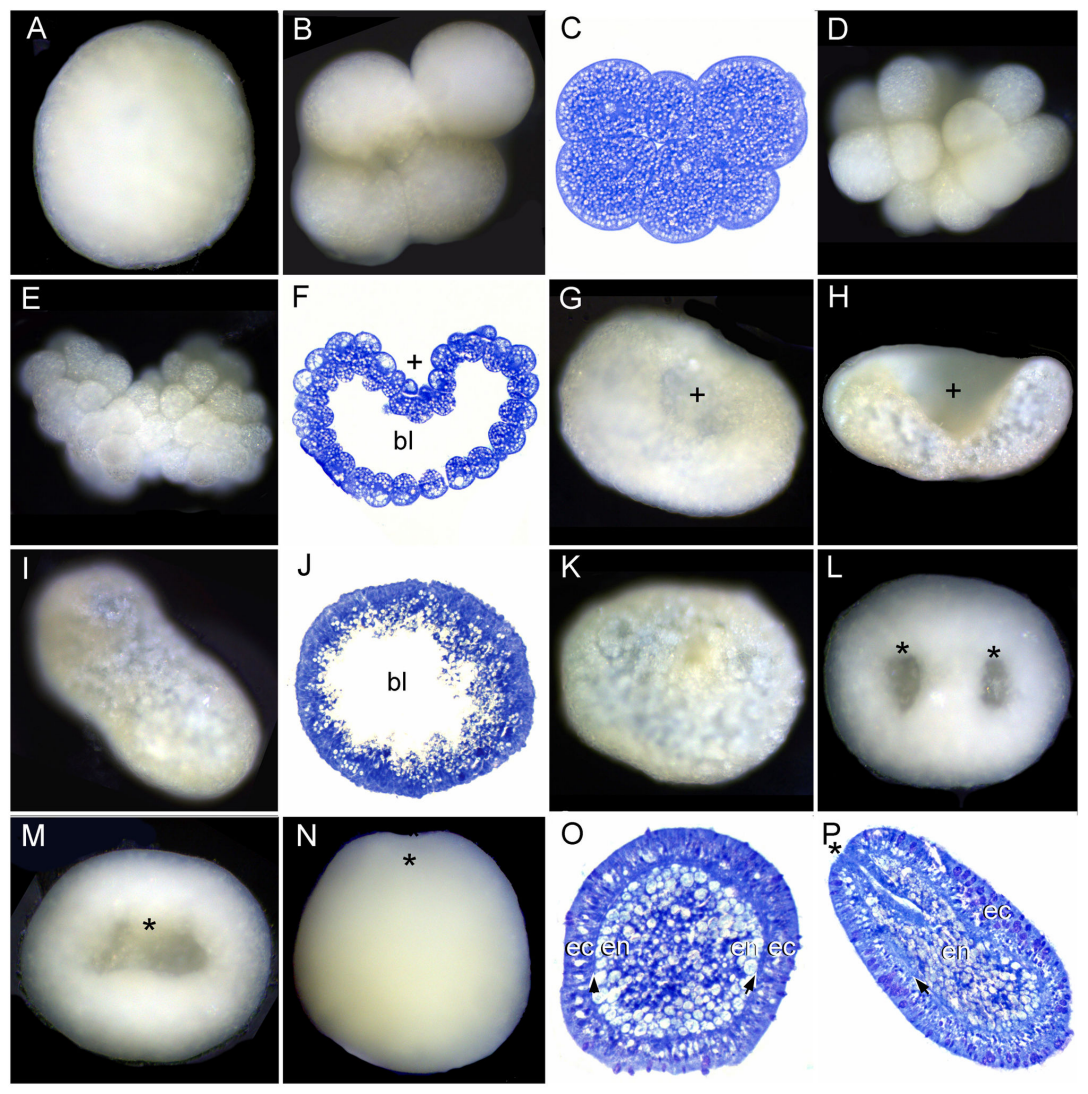
Development of *Platygyra contorta*. (A) Spawned egg. (B) 4-cell stage-the blastomeres are offset. (C) Section of slightly older embryo. (D) The 16-cell stage is much less regularly arranged than in the other species discussed here. (E) The embryo has started flattening and is now highly irregular in shape. (F) At a slightly older stage a blastocoel (bl) becomes apparent. The pseudo-blastopore is marked by a "+" in this and subsequent panels. (G) This stage corresponds to the prawn chip stage of *Acropora*, but it has a cushion shape due to the enclosed blastocoel. (H) Cell division has continued, leading to a smoother surface as the pseudo-blastopore deepens. Loosely consolidated lipid fills the area that will eventually be occupied by endoderm. (I-K) Embryos of this species are often highly variable in shape. At this stage lipid and cells are starting to move inward to fill the blastocoel (bl). (L, M) At gastrulation two separate pores appear initially (asterisks in L) and then expand and grow together until ultimately a single blastopore is formed (asterisk in M). (N) Eventually a slightly elongate planula is formed with the oral pore at one end (asterisk). (O) Transverse section of planula showing endoderm (en), mesoglea (arrows) and ectoderm (ec). (P) Elongate planula showing pharynx extending inward from the oral pore (asterisk).

*Oulastrea crispata* is known to be a hermaphrodite and it has also been reported to release planulae in Hong Kong [28,29]. However in the populations studied, some colonies released only eggs and others only sperm, so it may be gonochoric in Wakayama. The first cleavage resulted in two equal blastomeres (Figure 5A,B). Within 1 hour after the first cleavage, the second, third and fourth cleavages followed forming a spherical blastula (Figure 5C-G). After 3.5 hours a depression appeared in the side of the sphere, and the embryos gradually assumed a flattened shape (Figure 5H). After 6 hours the embryos swelled (Figure 6I-K), formed a hollow sphere with a smooth surface after 7 hours (Figure 6L), and became pear-shaped after 8-9 hours (Figure 6M). After 13 hours the embryos started moving, indicating that cilia had formed. After approximately 23 hours invagination started (Figure 6N). Invagination continued and the oral pore (asterisk) became apparent by 85 hr (Figure 6O,P) as the planula elongated and continued to develop.

#### *Platygyra contorta*

The first cleavage resulted in two equal blastomeres. Further cleavages produced an embryo of 8-32 cells after 4 hours (Figure 6A-D). This species has a very irregular shape from the morula (Figure 6D) to the cushion stage (Figure 6F,G), but from this irregular shape the embryos became roughly spherical, with the pseudo-blastopore apparent after 5 hours (Figure 6F-H, plus sign). The pseudo-blastopore then gradually disappeared (Figure 6I) and the embryo resumed a spherical shape after 14 hours (Figure 6J,K). A new invagination started at one point on the spheroid after 14.5 hours, followed shortly thereafter by a second, separate invagination (Figure 6L, asterisks), after which the two pores merged (Figure 6M, asterisk). These two pores may be similar to those of *Nematostella* of Figures 3D, 4C of Kraus and Technau [30]. The embryos started swimming after 19 hours (Figure 6N). The endoderm (en) of the planula is filled with lipid (Figure 6O) and remains that way as the planula elongates further (Figure 6P). Babcock & Heyward [27] show a section series for *Platygyra sinensis*, which complements the series shown here and shows a typical robust morphology in later stages. 

#### *Favites abdita* and *Favites pentagona*

**Figure 7:**
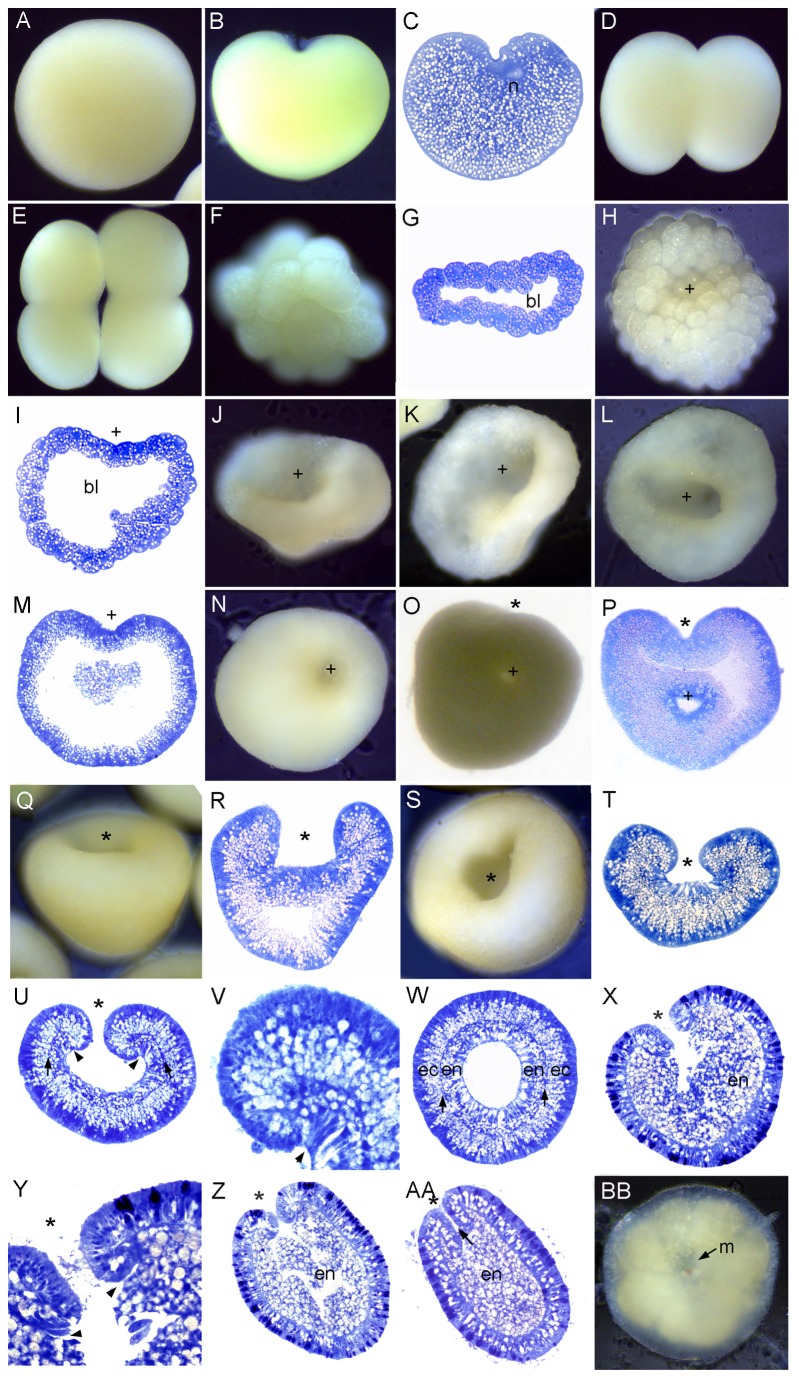
Development of *Favites abdita*. (A) Spawned egg. (B) This heart-shaped embryo is just beginning its first division. (C) Section of a heart- shaped embryo. The nucleus (n) appears to be just starting to divide. (D) A 2-cell embryo. (E) 4-cell stage (F) 16-cell stage. (G) The embryo has flattened and a blastocoel (bl in this and succeeding panels) has formed. (H-I) The embryo has now become cushion-shaped with a depression, the pseudo-blastopore (plus sign in this and subsequent panels) appearing in one side. (J-K) Embryos vary in shape as the pseudo-blastopore deepens. (L) The embryo swells, becoming more spherical, at the same time maintaining the pseudo-blastopore. (M) Section of a nearly spherical embryo. (N) Spherical embryo with the remains of the pseudo-blastopore. (O) A new invagination, the blastopore (asterisk in this and succeeding panels) starts in a different location from the pseudo-blastopore. (P) Section of an embryo comparable to O, showing that the two pores are quite distinct (labels as in O). (Q, R) Invagination has proceeded: the asterisk marks the blastopore. (S) The blastopore has now become smaller. (T) The blastocoel has now disappeared and cells at the margins of the invaginating tissue have taken on an elongate bottle shape. (U) The mesoglea is now clearly apparent, separating endoderm from ectoderm. (V) Higher magnification of U, showing highly elongated cells at the margins of the invaginating tissue. (W) Two germ layers, ectoderm (ec) and endoderm (en), are apparent surrounding the space that will eventually form the gastrovascular cavity. (X) Cellular differentiation is apparent in the ectoderm and lipid-filled endodermal cells have invaded the central cavity. (Y) Higher magnification of the oral pore region showing sharply invaginated margins of the pharynx (arrowheads). (Z) Section of the elongating planula showing the central cavity filled with lipid-containing endodermal cells (en). (AA) The pharynx (arrow) has elongated; (BB) Primary polyp immediately after settlement. The ectoderm is translucent while the endoderm is opaque white. The mouth (m) is central.

Figure 7 shows the development of *Favites abdita*. The development of *Favites pentagona* was generally similar to *F. abdita*, but with some differences as detailed below. The first cleavage resulted in two equal blastomeres. After 2 hours, the cleavage had produced 8-32 blastomeres (Figure 7A-F). Initially this was a solid mass of cells, but as the cell number increased the sphere of cells became hollow, forming a blastocoel (Figure 7G), and gradually flattening after 5 hours (Figure 7G,H). The shape of the embryo at this stage is more irregular in *F. pentagona* than *F. abdita*. A pseudo-blastopore then arose in the center of the disc shaped embryo as it again became more spherical (Figure 7H-N, plus sign). In *F. pentagona*, the embryo was completely spherical and the pseudo-blastopore began to disappear after 12-13 hours (in the interval 7L-M). Invagination of the blastopore started from after approximately 15 hours. In *F. abdita*, formation of the second pore (asterisk) had started before the first pore closed after 20-21 hours (Figure 7O,P). These results indicate that the first concavity, which could be mistaken for the blastopore but is not, does not become the mouth in *Favites*. *Favites pentagona* embryos started swimming ca. 15 hours and *F. abdita* embryos ca. 22 hours after the first cleavage (Figure 7Q,R). As invagination proceeded the blastocoel gradually disappeared (Figure 7S,T) and the embryo formed two germ layers, ectoderm and endoderm, separated by an acellular mesoglea (Figure 7U, arrows). As this occurred, invagination resulted in cells at the edges of the blastopore taking on an elongate bottle shape (Figure 7U-V, arrowheads). The lipid bodies became larger compared to previous developmental stages (Figure 7V). From this stage onward the main changes were elongation of the embryo, ingrowth of the pharynx, and differentiation of different cell types in the ectoderm (Figure 7W-AA). Mesenteries and mesenterial filaments were formed after 4 days (not shown) and after 7 days most planulae started to settle and metamorphose (Fig. 7BB). 

#### *Echinophyllia aspera*

**Figure 8:**
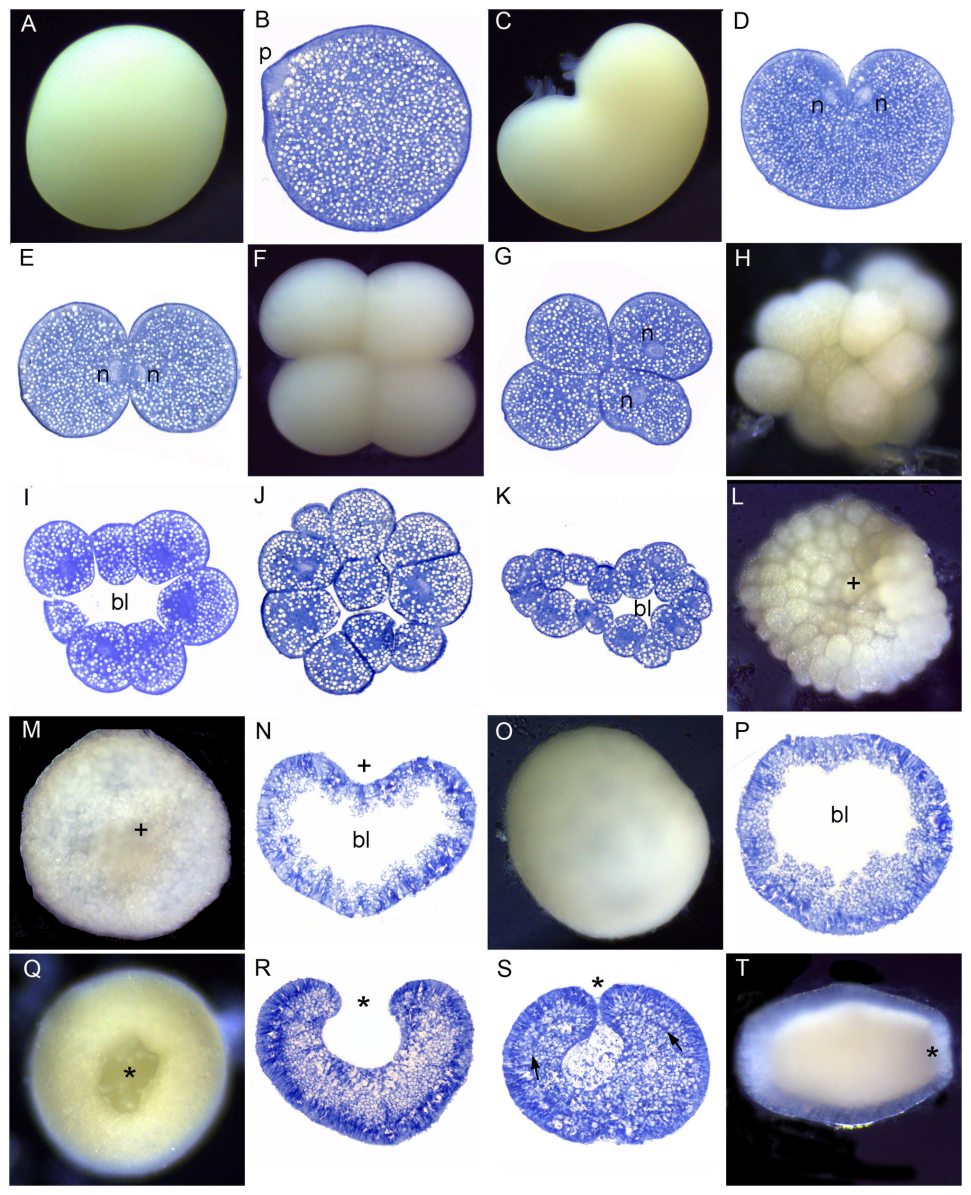
Development of *Echinophyllia aspera*. (A) A spawned egg. (B) A possible polar body pinching off. (C) The beginning of the cleavage furrow that will result in a 2-cell embryo; (D) Section of the stage shown in C. (E) Section of a 2-cell embryo. (F) At the 4-cell stage the cells are firmly attached to each other. (G) Section of a 4-cell embryo, with nuclei moving to the center of each cell. (H) 16-cell stage (I) Section of 16-cell stage with apparent blastocoel. (J) 16-32 cell stage (K) The embryo has started to flatten. (L) Cushion-shaped embryo (the plus sign marks the pseudo-blastopore in this and subsequent panels). (M) The return to a spherical state starts with swelling from the edge. (N) The pseudo-blastopore is still apparent. (O) A spheroidal embryo. (P) Section of an embryo comparable to O with material moving toward the blastocoel. (Q) Invagination of the blastopore/oral pore (asterisk) has begun. (R) Invagination has continued and the blastocoel has disappeared. (S) The cavity formed by invagination is being filled by the breakdown and migration of cells from the inner layer, and mesoglea formation has started (arrows). (T) Elongate planula.

The egg is roughly spherical with fine yolk granules evenly distributed (Figure 8A-B). The first cleavage resulted in a heart-shaped embryo (Figure 8C,D) which then divided to form 2 equal blastomeres (Figure 8E). After 3 hours the embryo consisted of 8-32 cells (Figure 8F-J). From approximately the 16-cell stage the cells are arranged in a hollow sphere (Figure 8J), which became flattened after 7 hours as the pseudo-blastopore developed and then started to disappear again (Figure 8K-N). The embryo then swelled and became spheroidal (Figure 8O). The embryo continued as a hollow spheroid for about 5 hours starting approximately 8 hours after the first cleavage (Figure 8P) and gradually became spherical after 14 hours. Blastopore formation commenced by 15 hours (Figure 8Q,R) and the embryos started swimming at about this stage. The blastopore then began to close but never disappeared, eventually becoming the mouth. Mesoglea developed, and lipid was released into the interior (Figure 8R,S). The embryo then elongated into a typical planula shape, and was swimming strongly by the stage shown in Figure 8T.

#### *Goniastrea favulus*

**Figure 9:**
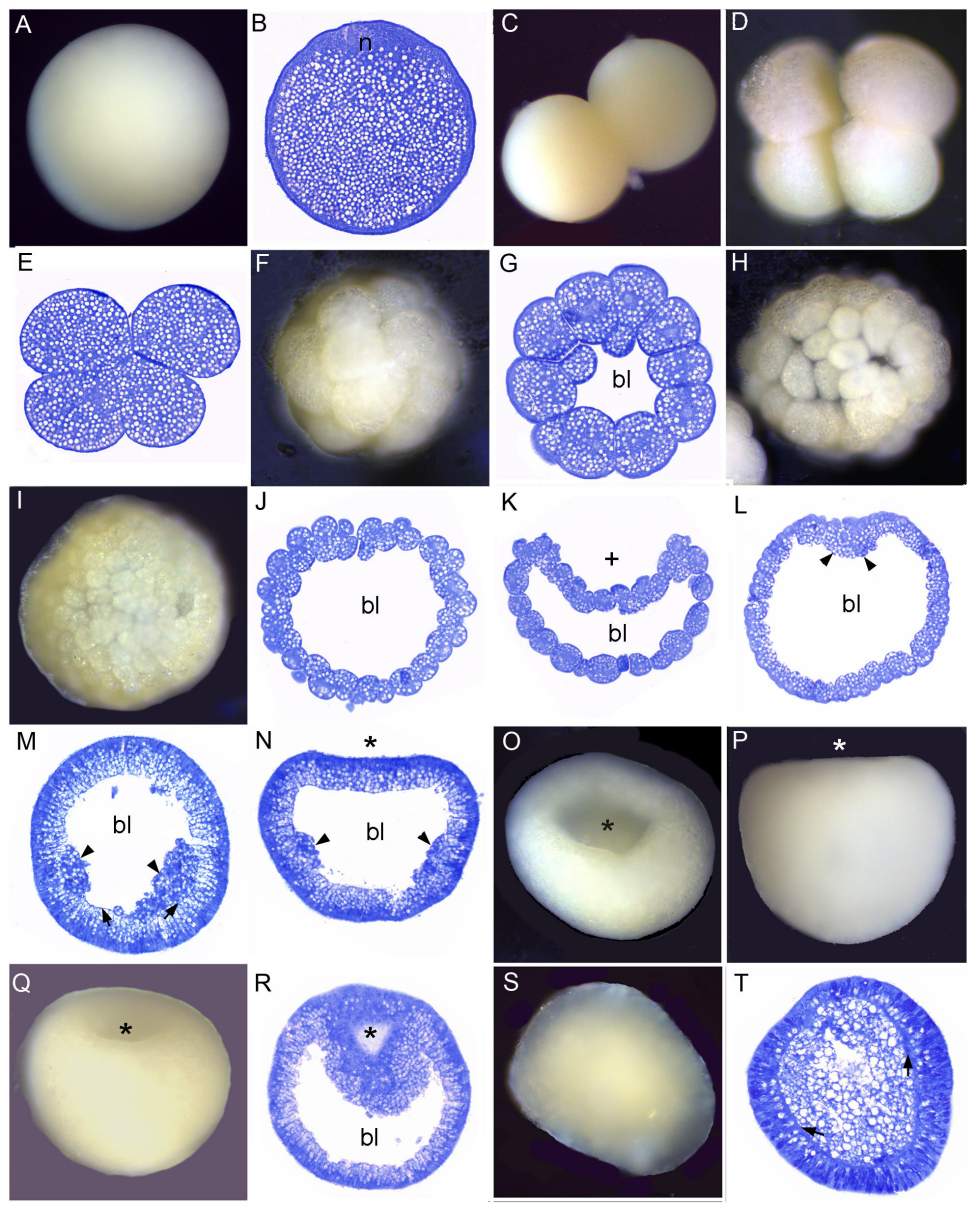
Development of *Goniastrea favulus*. (A) Spawned egg. (B) There is no lipid in the immediate vicinity of the nucleus. (C) 2-cell stage. (D) 4-cell stage. (E) Section of the 4-cell stage. (F) At the 16-cell stage the cells are organized into a sphere; (G) A section of the embryo at this stage reveals that the sphere is hollow, and that the cells surround a central blastocoel (bl, in this and succeeding panels). (H) 32-cell stage. (I) A spherical blastula. (J) Section of an embryo comparable to that shown in I. (K) The pseudo-blastopore is now apparent. (L) The embryo is spherical and material is starting to move into the blastocoel at one point (arrowheads). (M) Cell division has continued, material is moving into the blastocoel (arrowheads) and mesoglea is beginning to form (arrows). (N) Invagination of the blastopore (asterisk in this and subsequent panels) has started and there is material moving into the blastocoel from the sides. (O) The blastopore/oral pore has now formed a significant depression as the embryo resumes its formerly spherical shape (P). (Q-R) The oral pore shrinks in diameter as the embryo begins to elongate. (S) Planula. (T) Section of a planula showing the central area now filled with lipid and delimited by a well formed mesoglea (arrows).

No mucus egg coat was seen and the first 2 blastomeres were equal in the *Goniastrea favulus* population that we observed (Figure 9A-C). This contrasts to the same species on the Australian Great Barrier Reef, where a mucus egg coat is present and division is unequal [27]. Subsequent cleavages followed (Figure 9D-F), resulting in a hollow spherical embryo (Figure 9G,H) four hours after the first cleavage. The embryo then gradually assumed an irregular flattened shape (Figure 9I-K) as the cell surface became smoother due to continuing cell division. Figure 9K resembles the prawn chip stage in *Acropora* (Hayashibara et al., [14]; Ball et al., [15]) but is not nearly as flat, as this embryo contains a hollow blastocoel (bl) as well as the pseudo-blastopore (plus sign). By ca. 12 hours after the first cleavage the embryos again formed hollow spheres (Figure 9L,M). At about this stage, material began moving into the blastocoel (Figure 9M). At approximately 14-16 hours the embryos again became flatter, marking the start of blastopore formation (Figure 9N, asterisk). Two lateral areas of ingressing material are apparent in both Figure 9M and 9N. Whether this material is cellular, and thus possibly indicative of bipolar ingression, cannot be resolved by the methods used in this paper. However, the section shown in Figure 9R seems more consistent with gastrulation by invagination in that the majority of the internalized material seems to be associated with the blastopore. The embryos started swimming approximately 18 hours after the first cleavage (Figure 9O). Development proceeded (Figure 9P-R), and two germ layers, ectoderm and endoderm, were formed before the planula stage (Figure 9S-T) in which there is an obvious mesoglea (Figure 9T, arrows).

#### *Dipsastraea speciosa*

**Figure 10:**
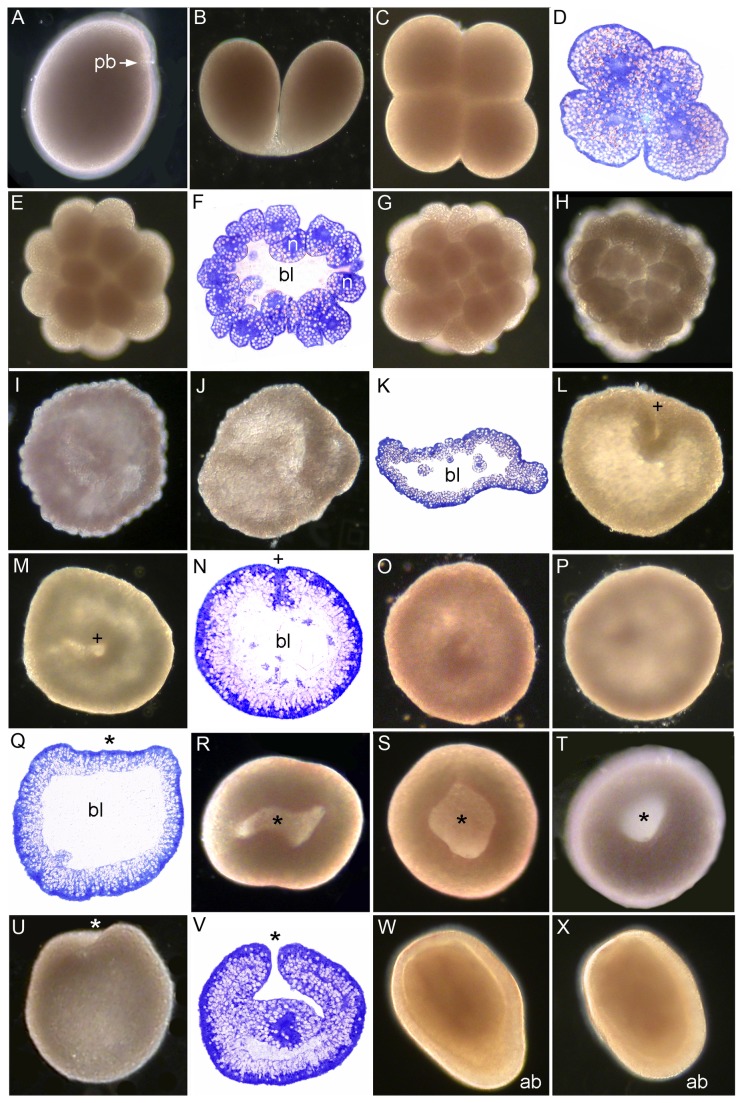
Development of *Dipsastraea *(Favia) *speciosa*. (A) Spawned egg, showing polar body (pb). (B) 2-cell stage, the blastomeres are almost fully separated. (C-D) 4-cell stage. The cells are tightly adherent. (E) 16-cell stage. (F) Section of 16-cell stage with blastocoel (bl in this and subsequent panels). Nuclei (n) occupy a substantial portion of the volume of each cell. (G) 32-cell stage. (H) The embryo has now started to flatten. (I-K) Further cell division and flattening have resulted in an embryo comparable to the prawn chip stage of *Acropora*, but for the presence of a significant blastocoel (bl). (L) The pseudo-blastopore has appeared (plus sign). (M) The embryo has now become spherical with a smooth surface except for the invagination associated with the pseudo-blastopore. (N) Section of spherical embryo with the pseudo-blastopore; within the periphery of the embryo is a layer of lipid globules. The blastocoel is starting to fill. (O, P) The pseudo-blastopore has now closed. (Q) This section reveals a flattening where invagination of the blastopore is about to begin (asterisk). (R) Invagination of the blastopore has now started. (S-T) The blastopore has first become circular and then started to shrink in diameter. (U) The embryo has now begun to elongate in the oral-aboral axis. (V) This section shows the blastopore closing and two germ layers being formed. (W) A pear shaped planula with aboral end (ab in this and the next figure) becoming thicker. (X) Barrel-shaped planula.

A polar body (pb, Figure 10A) started to appear in several eggs approximately 1 hour after spawning while the eggs were still spheroidal. Next the polar body came off and the egg became spherical (not shown). The first cleavage resulted in two equal blastomeres (Figure 10B) and the second cleavage was observed approximately 1 hour after the first (Figure 10C,D). Cleavage proceeded and the embryo became hollow and flattened (Figure 10E-K). Next it became more spherical and developed a concavity, the pseudo-blastopore, in one side (Figure 10L, plus sign) which remained (Figure 10M-N, plus sign) for four hours. This concavity disappeared before the start of a second invagination after 13 hours (asterisk in Figure 10Q and subsequent figures), which established an irregularly shaped, elongate cavity which gradually became round (Figure 10S). The embryo began to swim in the upper or intermediate water layer of the container in which it was held at about 14-17 hours, while the cavity was still opening. The pore then gradually reduced in size (Figure 10T,U) and there is evidence of endoderm formation by unipolar ingression (Figure 10V). The pore eventually becomes the mouth of the planula larva. After approximately 24 hours some of the planulae began swimming toward the bottom of the bowl, assuming a pear (Figure 10W) or barrel shape (Figure 10X) after 39 hours.

#### *Phymastrea valensiennesi*

**Figure 11:**
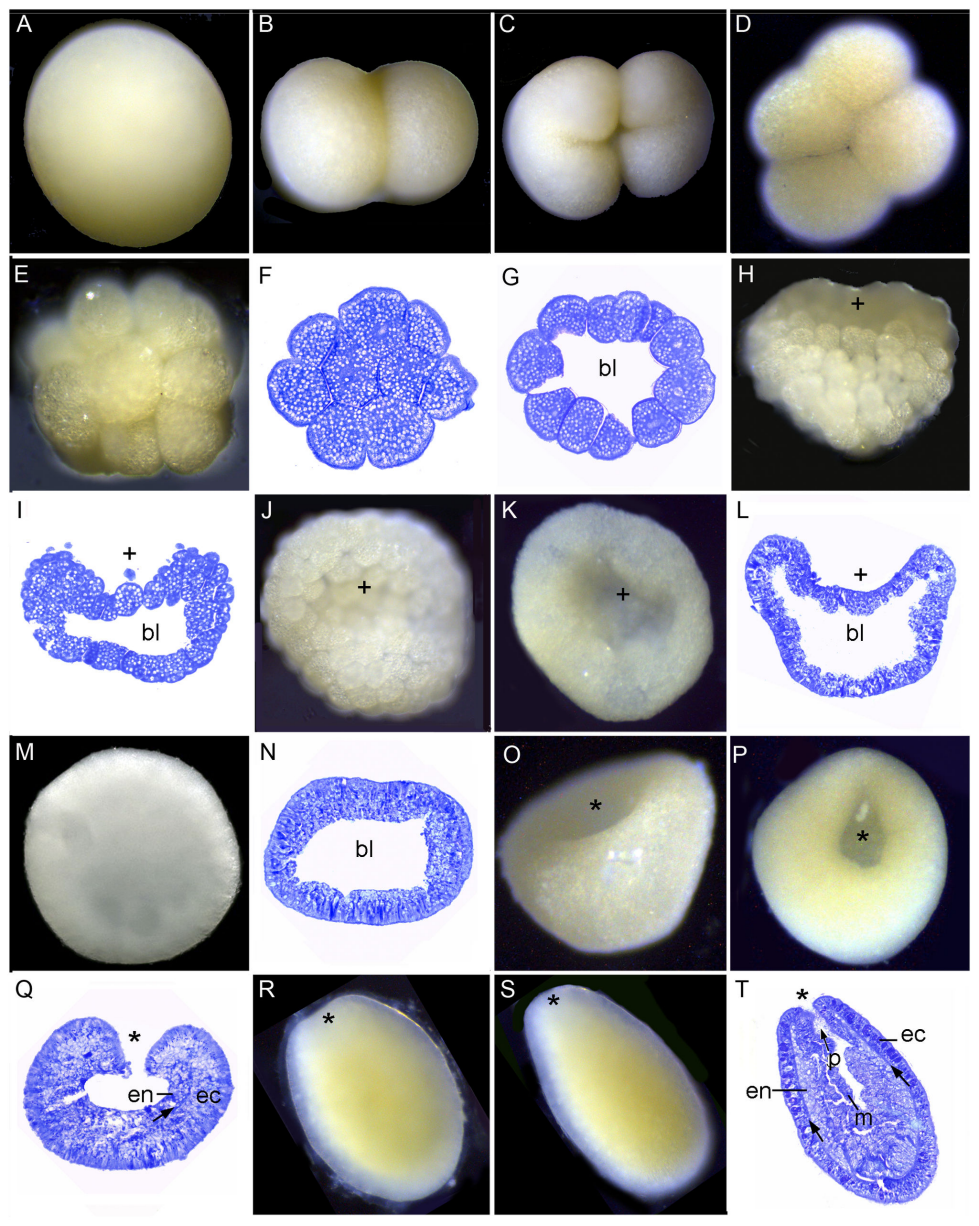
Development of *Phymastrea valenciennesi*. (A) Spawned egg. (B) 2-cell stage. (C) The first and second cleavage furrows both start from the animal pole. (D) 4-cell stage. (E) The 16-cell stage is roughly spherical. (F) A section of the 16-cell stage reveals that it is a solid mass of cells. (G) By the 32-cell stage a blastocoel (bl) has developed. (H) A pseudo-blastopore (plus sign) has developed in the side of the formerly spherical embryo. (I) Section of an embryo comparable to that shown in H. (J-K) Older embryos, looking down on the deepening pseudo-blastopore. (L) Section of an embryo comparable to that shown in K. (M) The pseudo-blastopore has disappeared and the embryo has expanded to form a spheroid, as shown in N. (O) Formation of the blastopore (asterisk in this and later panels) has begun. (P) The blastopore/oral pore begins to close. (Q) Invagination is proceeding, the blastocoel has disappeared, mesoglea is forming (arrow), and distinct endoderm (en) and ectoderm (ec) are becoming apparent. (R-S) The planula gradually elongates and the translucent ectoderm is clearly differentiated from the opaque cream-colored central endoderm. (T) A longitudinal section of a mature planula reveals a well differentiated ectoderm (ec) separated from a still lipid-filled endoderm (en) by a well-developed mesoglea (black arrows). The pharynx (p) and well-differentiated mesenteries (m) are also apparent.

The first cleavage resulted in two equal blastomeres (Figure 11A,B). Cleavage proceeded and the embryos reached the 8-32 cell stage after 3 hours (Figure 11C-F), by which time a blastocoel had developed (Figure 11G). A pseudo-blastopore (plus sign) appeared and continued to deepen as cell division continued (Figure 11H-L). It then gradually disappeared (Figure 11M-N), before the blastopore opened as the embryo became bowl-shaped (Figure 11O, asterisk) and swimming began, indicating the development of cilia approximately 19 hours after first cleavage. The blastopore deepened and mesoglea formation began after 60 hours (Figure 11P,Q). The embryo then elongated, assuming a typical planula shape (Figure 11R, S). A section of the mature planula shows that the mesenteries had formed and the mesoglea was strongly developed (Figure 11T).

## General Discussion

### Polar bodies and *Symbiodinium*

A polar body (e.g. *Montipora*, Figure 3C,D, *Dipsastraea*, Figure 10A) could be observed within 0.5 - 1 h after spawning in all species except *O. crispata* and *P. decussata*, although whether it was the first or second polar body is unknown. Polar body formation is a defining feature of the animal pole [31] and when zygotes were continuously observed following polar body release this was also the end of the zygote where cleavage was initiated, consistent with its identification as the animal pole.

*Montipora* is the only genus considered here which transmits its *Symbiodinium* vertically (through the egg), and we found that *Symbiodinium* enter the oocyte between 8 and 32 hours before spawning. After spawning *Symbiodinium* were not found immediately adjacent to the site of polar body emergence (Figure 3C) and were sometimes unevenly dispersed (Figure 3H), although there was no consistent pattern of spatial restriction except that essentially all had moved to the endoderm before settlement. 

### Cleavage

Cleavage was holoblastic in all species, even though yolk is abundant in all except *Oulastrea crispata and Pavona Decussata*, the two species with the smallest eggs (Table 1), and with embryos that sank. *Pseudosiderastrea* embryos also sank, although normally they would be caught in the mucus net, and started swimming from 3 days after spawning. In contrast, embryos of the other species with similar-sized eggs did not sink and started swimming much earlier. In histological sections the lipid-filled cells in *Oulastrea, Pavona* and *Pseudosiderastrea* are very small compared to the other studied species. In *Pseudosiderastrea* the lipid droplets were very small during early cleavage stages but during gastrulation larger droplets gradually appeared (Figure 1K), presumably by fusion of the small. Ability to float may be related to wax ester content and *Oulastrea* has little wax ester and *Pseudosiderastrea* less, compared to broad dispersal genera like *Acropora* [32]. It is not known whether there is a relationship between the amount of stored lipid and the time at which swimming behavior starts.

In all species the first cleavage furrow is initiated at the animal pole, creating a heart-shaped zygote (Figures 1B; 3F; 7B,C; 8C,D; 10B). Cleavage then splits the egg almost symmetrically at 2 h post fertilization (e.g. Figures 4B; 5B; 7D; 8E; 9C; 11B). The second cleavage furrow is also initiated at the animal pole. The first two blastomeres are slightly offset; thus the cleavage plane for each blastomere is not at right angles to the furrow of the first cleavage. Four blastomeres are produced approximately 3 h after the first cleavage (Figures 1C,D; 2G; 3G; 4D,E; 5C; 6B; 7E; 8F,G; 9D,E; 10C,D; 11C,D). Thereafter, no consistent pattern was detected. From approximately the 32-cell stage, the complex corals (with the exception of *Pavona*) and robust corals follow somewhat different developmental paths in that the complex corals *Pseudosiderastrea, Galaxea* and *Montipora* and *Acropora* [14,16,19] pass through an expanded stage consisting of a cellular bilayer lacking a blastocoel (Figures 1,2,3). Therefore the two clades will be considered separately from this point.

An important question relating to cnidarian gastrulation, which has plagued investigators for many years (summarized in [18,33]), is the nature of the material deposited in the blastocoel of those species which form one. It is clear that lipid moves from being relatively evenly distributed in early blastomeres to being concentrated in the endoderm in later stages, but the mechanism by which this occurs is unclear. This is also the fate of maternally seeded *Symbiodinium*, which go from an even distribution in early blastomeres to being restricted to endoderm in later stages. However, the extent to which pre-existing cells are migrating into the endoderm, as opposed to being added there by cell division from the ectoderm is unclear. This topic has only been investigated with modern methods in corals by Marlow and Martindale [18], who found that in the case of *Fungia scutaria* lipid-rich cells migrated into the blastocoel, while in *Pocillopora meandrina* it was membrane-bound cellular fragments.

### Formation of two germ layers in complex corals

**Figure 12:**
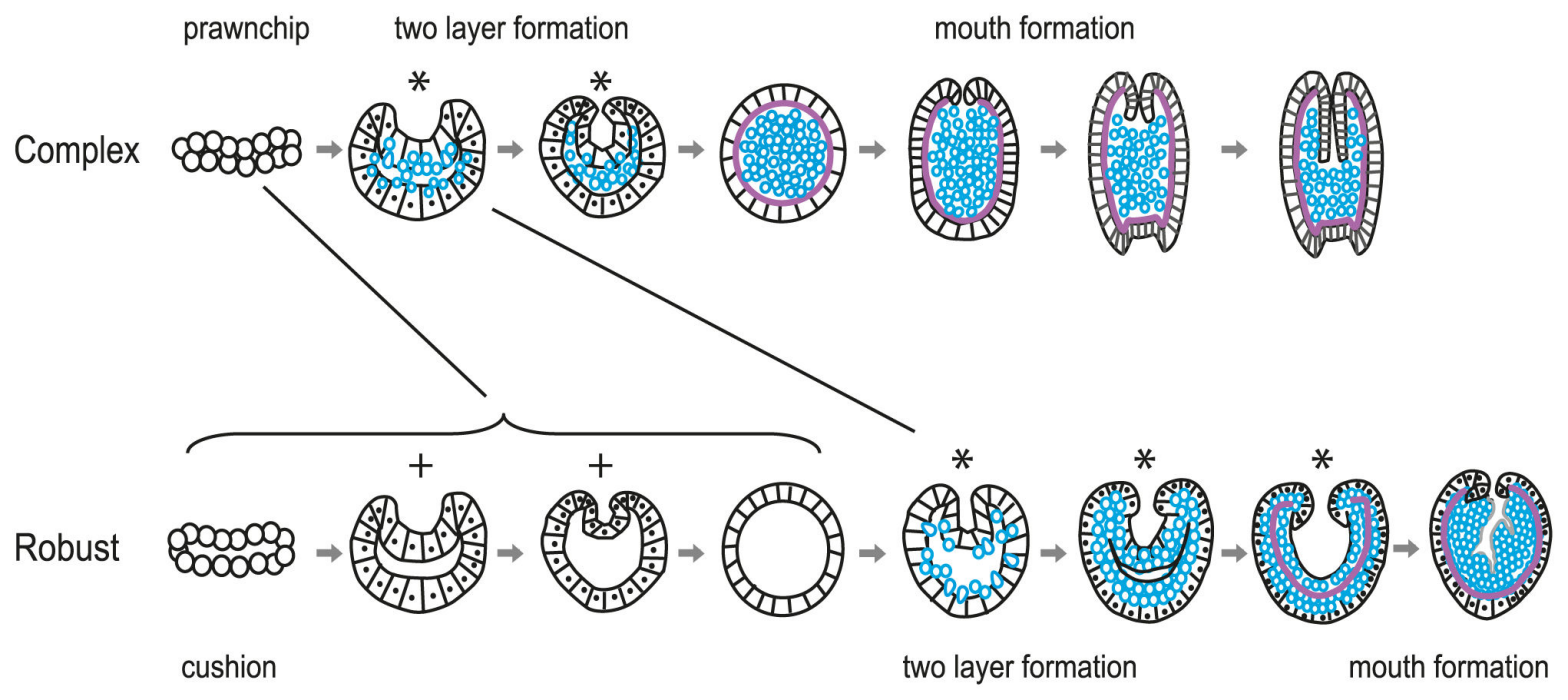
Diagrammatic representation of the two extreme forms of coral development. (A) Early in their development *Acropora* *spp.* embryos pass through a prawn chip stage consisting of an extended cellular bilayer lacking a blastocoel [14, 15, 19]. Through changes in cell shape this extended sheet of cells shrinks in diameter, thickens and the sides bend inward, forming a bowl-shaped embryo. The ultimate result of these movements is that the cells lining this concavity are overgrown by the outer cells, resulting in an outer sphere of ectoderm surrounding an inner mass consisting of lipid granules, cellular fragments and cells. This outer sphere is complete, with no trace of a pore to be seen. We regard this process as gastrulation and the initial pore as the blastopore. Whether or not this interpretation is accepted this process is markedly different from that shown in (B) which is that seen in robust corals such as *Goniastrea favulus*. Rather than a spatially extended prawn chip lacking a blastocoel, these corals pass through a cushion stage, which is flattened but always retains a blastocoel. This cushion then rounds up and develops a depression in its side which we have termed a "pseudo-blastopore", but no material enters the blastocoel. The pseudo-blastopore then disappears, the embryo rounds up again, and then a second pore, the true blastopore, appears, this time associated with the passage of material into the blastocoel. In such corals the blastopore remains open and transitions seamlessly into oral pore/mouth.

The embryos of *Galaxea* (Figure 2J-N) and *Montipora* (Figure 3K-N) pass through a flattened bilayered stage similar to that seen in *Acropora* spp. [14,17,34]. In *Pseudosiderastrea* this bilayer takes on a complex morphology, winding back and forth upon itself and remaining spherical rather than flattening to form a characteristic prawn chip stage such as that seen in *Acropora* spp. (Figure 1H-J). *Pavona*, in contrast, develops in a manner more similar to the robust corals, with which it will be described. In *Pseudosiderastrea, Galaxea* and *Montipora* the outer cell surface of the embryo gradually becomes smoother as the cells divide and become smaller in diameter (Figures 1I,J; 2J,K; 3L-N). Then the blastula gradually becomes thicker, as the cells elongate at right angles to the flattened disc, and begins to become spherical as the sides fold inward to form the blastopore (Figures 1J-M; 2J,K,N,O; 3N,O,P). The pore remains visible only in *Montipora* (Figure 3O-Q). It should be apparent from the above description that the process by which the embryo makes the transition from the prawn chip to the spherical gastrula remains unclear at a mechanistic level, and will only become apparent through the use of cell marking techniques. The stages described above are schematically summarized in Figure 12A. 

### Mouth formation by invagination in complex corals

The now spherical larvae develop cilia on the outer surface and start rotary swimming (Figures 2P; 3Q), with the exception of *Pseudosiderastrea* which remains in the mucus net and starts swimming from 3 days after spawning. The pore remains continuously visible in *Montipora*, and can be seen from the outside under the microscope. In all species except *Pavona*, the outer cells become columnar, forming a single layer of epidermis (Figures 1N; 2N; 3P) surrounding a central area containing lipid bodies and cellular fragments. Whether new endodermal cells are being formed or existing cells are expanding due to lipid uptake remains to be established and will only be resolved by a mixture of cell marking experiments and advanced histochemical and microscopical techniques, as pioneered by Marlow and Martindale [18] for robust corals. However, such experiments will not be easy for mass spawning complex corals because these stages are only available for a few hours each year. In spherical swimming planulae and pear-shaped planulae the boundary between the inner and outer germ layers becomes clear, indicating mesoglea formation (Figures 1O; 3Q). The timing of mesoglea formation is later in *Pseudosiderastrea* than the other studied complex corals. The oral pore (mouth) forms by invagination and the larvae elongate further in *Pseudosiderastrea* and *Montipora* (Figures 1P; 3T).

### Formation of two germ layers by invagination in robust corals

The robust corals and *Pavona* differ from the complex corals, in that a blastocoel is formed after the early cleavage divisions (Figures 4H; 6F; 7G; 8I; 9G, 10F; 11G). Next, the spherical blastula flattens to a concave cushion shape (Figures 4L,M; 6F; 7H,I; 8L; 9K; 10H-K; 11H-J). At this stage the embryo resembles the gastrulating "fat donut" stage of *Acropora millepora* [15]. However, in the robust corals there is only a single cell layer surrounding a blastocoel and this stage is actually more comparable in developmental timing and topology to the prawn chip stage of complex corals. The concavity, which appears to be a common feature of robust corals at this stage, is therefore not a blastopore. The functional significance of this pseudo-blastopore is unclear since in all cases the embryo subsequently resumes a more spherical shape. Lipids and cell fragments then start to move into the blastocoel. This is followed by invagination (Figures 5N; 6L-M; 7O,P; 8Q,R, 9N,O; 10Q,R; 11O,P), which leads to creation of the endoderm. In *Favites*, the pseudo-blastopore persists and invagination to form the endoderm occurs from a different position in the side of the cushion-shaped embryo (Figure 7O,P). As invagination proceeds (Figures 5N-P; 6N; 7R-Z; 8R,S; 9Q-S; 10U-W; 11Q,R), the embryo develops cilia, begins swimming, and the pharynx is formed (Figures 5P,6P, 7AA.,11T).

## Conclusions

As indicated above, the descriptive developmental data available for corals are quite limited, considering the number of species and their morphological diversity. So, our first goal in writing this paper was to provide information on the development of a number of species additional to those which have previously been described. Some of the accounts that we have provided are far from complete, but hopefully this will spur others on to fill in the missing details. A second goal was to see whether the prawn chip stage typical of the *Acropora* species that we had previously studied (e.g. 15-17,19) was a characteristic feature of the development of complex corals. We found that this was not the case, since the genus *Pavona*, which is listed among the complex corals in all recent phylogenies [2-5] lacks a prawn chip and has a well developed blastocoel comparable to that of the robust corals.

Figure 12 summarizes the divergent patterns of development seen in complex (as exemplified by *Acropora*) and robust corals (as exemplified by *Goniastrea*), as well as illustrating some of the descriptive terms used in the text. A major difference between the two groups, which to the best of our knowledge has not previously been reported, is the existence of the pseudo-blastopore. This is an initial invagination, which instead of leading directly into gastrulation as it does in the complex corals, is followed by a return to a spherical shape before a second invagination forms as a part of the gastrulation process. In most cases there is no temporal overlap between the two invaginations, and the spatial relationship between them is unclear. However, in *Favites* the blastopore, which gives rise to the mouth, forms while the pseudo-blastopore is still present, showing that in this species, at least, the two are spatially distinct. As is the case for the prawn chip morphology of complex corals, the functional significance of the pseudo-blastopore is unknown. 

We have tried to be conservative in attributing a mechanistic significance to the images of gastrulation shown in this paper. Certainly the majority could be interpreted as indicating that invagination plays a major role, but whether epiboly or other mechanisms are also involved remains to be determined by cell marking experiments and more detailed observation.

In an effort to broaden our survey of gastrulation patterns we turned to the literature, with the results summarized in Table 2. Some of the descriptions support a correlation between membership of the robust or complex group and pattern of development, while others, such as those of *Pocillopora*, are more equivocal. For some species the descriptions are not sufficiently complete for the type of early development to be unequivocally determined, but we have included them in an effort to make our survey complete. So, in the absence of other information, if a species forms a coeloblastula, it is likely to be a robust coral, while if it forms a prawn chip it is likely to be a complex coral. Nevertheless, as more species are described it may be that a continuum of degrees of development of the blastocoel will be discovered, with the well developed and persistent blastocoel of some robust genera (e.g. *Goniastrea*) at one end of the spectrum and the minimal blastocoel, associated with the prawn chip morphology of complex genera (e.g. *Acropora*), at the other.

**Table 2:** Gastrulation Patterns from the Literature.

	Family	Coeloblastula	Evidence	Reference
**Complex Corals**				
*Porites cylindrica*	Poritidae	no	Figure 1D,E	[25]**^*a*^**
*Leptosammia pruvoti**^b^***	Dendrophylliidae	no		[40]
*Astroides calycularis**^b^***	Dendrophylliidae	no		[41]**^*c*^**
*Monomyces rubrum**^b^***	Flabellidae	no		[42]
*Galaxea fascicularis*	Oculinidae	no	Figure 3	[24]
*Acropora digitifera*	Acroporidae	no	Figure 4, text	[19]
*Acropora florida*	Acroporidae	no	Figure 3, text	[14]
*Acropora hyacinthus*	Acroporidae	no	Figure 3, text	[14,19]
*Acropora intermedia*	Acroporidae	no	Figure 4, text	[19]
*Acropora millepora*	Acroporidae	no	Figure 4	[16]
*Acropora muricata*	Acroporidae	no	Figure 3	[24]
*Acropora nasuta*	Acroporidae	no	Figure 3, text	[14]
*Acropora pulchra*	Acroporidae	no	Figure 2, I, J	[34]
*Acropora secale*	Acroporidae	no	Figure 3, text	[14]
*Acropora solitaryensis*	Acroporidae	no	Figure 4, text	[19]
*Acropora tenuis*	Acroporidae	no	Figure 4, text	[19]
*Montipora digitata*	Acroporidae	no	Figure 2D,E,F,G	[25]
**Robust Corals**				
*Pocillopora damicornis**^c^***	Pocilloporidae	yes	Figure 2d	[43]
*Pocillopora meandrina**^c^***	Pocilloporidae	yes	Figure 5d	[18]
*Platygyra sinensis*	Faviidae	yes	Figure 3C	[27]
*Fungia scutaria*	Fungiidae	yes		[18]
*Astrangia danae*	Rhizagiidae	yes		[44]

The classification into robust/complex follows Kitahara et al [3]. ***^a^*** Figure 1 D,E of ref [25] shows a small blastocoel with a large amount of surrounding yolk. ***^b^*** more data needed. ***^c^*** Small blastocoel reported.

The present gaps in our knowledge of cnidarian development beyond the Scleractinia, heighten our interest in finding out more about embryonic development in presently uninvestigated groups. Critically important groups for which no information appears to be available include the Basal Scleractinia of Stolarski et al. [35], which are deep water solitary corals, the Antipatharia, and the Corallimorpharia. Based on present knowledge, patterns of cnidarian early development do not correlate consistently well with most current phylogenies. Thus, although the zooanthid *Palythoa tuberculosa* clearly has a "complex" type of gastrulation [36] consistent with the idea that this is the ancestral scleractinian mechanism of gastrulation, many anemones (Actinaria) form a coeloblastula [37], (reviewed in 38,39). Thus, there is still a great deal to learn about the evolution of early developmental patterns and their functional significance, both within the Scleractinia and in the Cnidaria as a whole. 

## Supporting Information

Figure S1 **Species studied in relation to phylogeny.** The coral species described in this paper provide good representative coverage of the complex (clade C, names in blue) and robust (clade D, names in red) clades as shown here, where they are overlaid onto the coral phylogeny of Kitahara et al. [3] which is based on the sequence of the mitochondrial CO1 gene.
(TIF)
