# Supplementary figures and images for: Comparative Embryology of Eleven Species of Stony Corals (Scleractinia)
Source: PLoS One. 2013 Dec 18;8(12):e84115. doi: 10.1371/journal.pone.0084115 (PMC3867500; doi:10.1371/journal.pone.0084115)

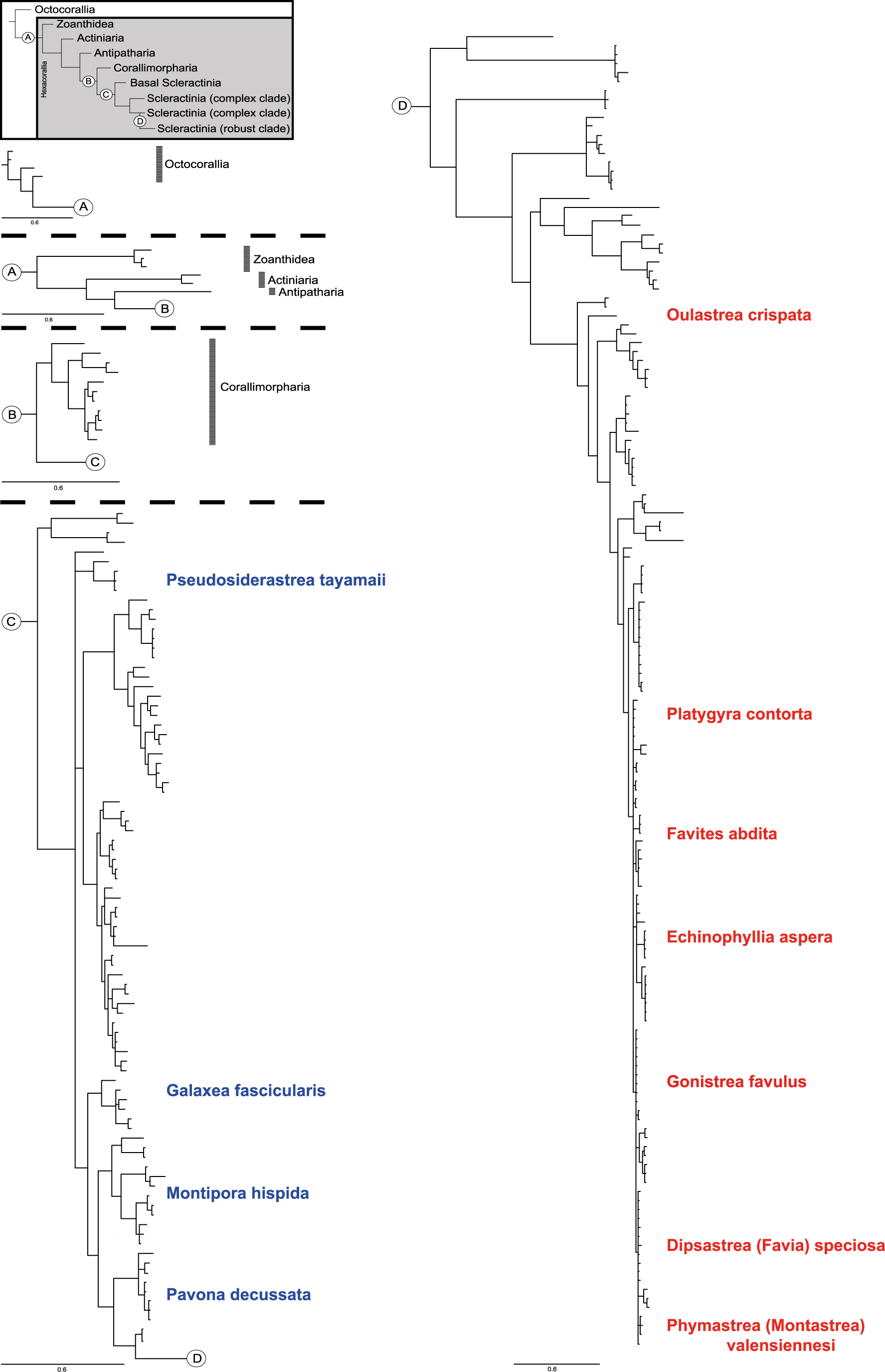

Supplement: Figure S1 — Species studied in relation to phylogeny. The coral species described in this paper provide good representative coverage of the complex (clade C, names in blue) and robust (clade D, names in red) clades as shown here, where they are overlaid onto the coral phylogeny of Kitahara et al. [3] which is based on the sequence of the mitochondrial CO1 gene. (TIF) [file pone.0084115.s001.tif]
